# Comparison of lipopolysaccharide-mediated peripheral blood mononuclear cell activation between Brahman and Brahman × Thai native crossbreed cattle

**DOI:** 10.14202/vetworld.2024.804-810

**Published:** 2024-04-10

**Authors:** Piyarat Srinontong, Worapol Aengwanich, Sattabongkod Somphon, Siriyakorn Khonwai, Thanasorn Nitsinsakul, Zhiliang Wu, Thanyakorn Chalalai, Bhuripit Saraphol, Wilasinee Srisanyong

**Affiliations:** 1 Faculty of Veterinary Sciences, Mahasarakham University, Mahasarakham 44000, Thailand; 2 Bioveterinary Research Unit, Faculty of Veterinary Sciences, Mahasarakham University, Mahasarakham 44000, Thailand; 3 Stress and Oxidative Stress in Animal Research Unit, Faculty of Veterinary Sciences, Mahasarakham University, Mahasarakham 44000, Thailand; 4 Department of Parasitology and Infectious Diseases, Gifu University Graduate School of Medicine, Gifu 5011194, Japan; 5 Department of Veterinary Technology, Faculty of Agriculture Technology, Kalasin University, Kalasin 46000, Thailand

**Keywords:** Brahman cattle, lipopolysaccharide, oxidative stress, peripheral blood mononuclear cells, Thai native crossbreed cattle

## Abstract

**Background and Aims:**

Lipopolysaccharide (LPS) is a robust endotoxin known to activate the immune system in cattle. The objective of this study was to investigate the effect of LPS on the morphology, cell viability, malondialdehyde (MDA), nitric oxide (NO), and total antioxidant capacity (TAC) of peripheral blood mononuclear cells (PBMCs) in Brahman and Brahman × Thai native crossbreed cattle.

**Materials and Methods:**

PBMCs were isolated from Brahman and Brahman × Thai native crossbreed cattle and treated with 0, 0.1, 1, and 10 μg/mL *Escherichia coli* LPS, respectively. Morphological changes in PBMCs were assessed at 24 and 48 h. In addition, we measured PBMC cell viability, MDA, NO, and TAC.

**Results:**

LPS stimulation caused cell deformation and partial PBMC area enlargement, but there were no differences between Brahman and Brahman × Thai native crossbreed cattle. Stimulation at all levels did not affect the viability of PBMCs (p > 0.05). MDA and NO levels were significantly higher in Brahman cattle than in Brahman Thai native crossbred cattle (p < 0.05). TAC was significantly higher in Brahman × Thai native crossbred cattle than in Brahman cattle (p < 0.05).

**Conclusion:**

Immune cells of crossbreed cattle have a higher activation response to LPS than those of purebred cattle, and native crossbreed beef cattle have a higher antioxidant capacity than purebred beef cattle. This result may explain why hybrid cattle of indigenous breeds are more resistant to disease than purebred cattle.

## Introduction

Beef is a high-quality source of protein with high global demand [1]. Beef production plays a vital role as a source of food, income, employment, and investment opportunities in Thailand [2]. The growing world population is exerting increased pressure on the cattle industry, among other things. Intensification and expansion of beef production inevitably increase the risk of spreading infectious diseases and exacerbate them. Therefore, an improved understanding of the immune function of cattle is needed to provide optimal tools to combat existing and future pathogens and to improve food security [3]. The immune system is composed of cells capable of detecting dangerous signals emanating from foreign pathogens. In addition, this system has the ability to rapidly diversify its response depending on the nature of the pathogen by utilizing a large number of immune responses. Livestock breeds have been selected for their high production and good immune response. Recent studies have demonstrated that it is possible to identify and selectively breed livestock with an inherent ability to make superior immune responses that can reduce disease occurrence and increase farm profitability [4]. In Thailand, there is a high demand for high-quality beef. This has led to an increase in the commercial raising of beef cattle [5]. Thai native cattle, Brahman cattle, Brahman crossbreeds, and fattening beef cattle are popularly raised in Thailand. They are adaptable to the local environment and resistant to pathogens [6, 7].

Lipopolysaccharide (LPS) is an essential component present in the cell walls of Gram-negative bacteria. It plays a pivotal role in inducing host inflammatory responses and diverse biological immune reactions, such as fever and septic shock [8], and may cause animal mortality [9]. Exposure to LPS initiates a cascade of events. LPS initially binds to cluster of differentiation 14 (CD14) and the myeloid differentiation-2/toll-like receptor 4 receptor complex, which is expressed in macrophages, monocytes, and dendritic cells [8]. This activation leads to the production of proinflammatory mediators against the infection. The effects of LPS on the immune system will be understood in terms of the mechanisms of infectious diseases and host self-defense [10].

Oxidative stress results from increased production of free radicals in animal body cells. By releasing free radicals, the body is subjected to chemical stress because it cannot neutralize or eliminate the free radicals [11]. Malondialdehyde (MDA) is an end product of the peroxidation of polyunsaturated fatty acids. Elevated MDA level is widely known as a biomarker for indirect measurement of oxidative stress [12]. Nitric oxide (NO) is a ubiquitous gaseous molecule that can freely pass across cell membranes. Endogenous NO is an important effector and signal transduction molecule in numerous cellular processes involved in physiological states. However, higher concentrations of NO can promote oxidative stress due to the different cellular properties and targets, leading to cytotoxicity [13]. Some commonly used antioxidant biomarkers, including total antioxidant capacity (TAC), remain the most widely used methods to quantify the oxidant-buffering capacity in a sample [14].

Zhao *et al*. [15] reported the effects of LPS on bovine mammary epithelial cells. LPS treatment increased NO and MDA production but did not affect cell viability at concentrations between 2 and 100 μg/mL. Li *et al*. [16] demonstrated that LPS exposure decreased TAC, superoxide dismutase, and catalase levels in bovine mammary epithelial cells. Zhao *et al*. [17] found that LPS elevated early apoptotic rates in bovine oocytes. The majority of previous studies have focused on the effects of LPS on cattle body cells.

However, there is limited information on the effects of LPS on immune system cells in cattle. We hypothesized that the responses of immune system cells to LPS stimulation may differ among different breeds of beef cattle raised in Thailand. Therefore, this study aimed to examine the effect of LPS on peripheral blood mononuclear cells (PBMCs) morphology, cell viability, MDA, NO, and TAC of Brahman and Brahman × Thai native crossbreed cattle. The knowledge gained from this study will provide a basis for understanding the effect of LPS, a product of pathogenic bacteria, on the immune system cells of beef cattle, including the susceptibility of different breeds of beef cattle to pathogens and their resistance.

## Materials and Methods

### Ethical approval

The Institution’s Ethics Committee on Animal Experimentation of Mahasarakham University approved the experimental procedures (IACUC-MSU-30/2023).

### Study period and location

This study was conducted from December 2022 to September 2023 at the Faculty of Veterinary Sciences, Mahasarakham University, Mahasarakham, Thailand.

### Animals

The study used six female cattle comprising three Brahman cattle and three Brahman × Thai native crossbreeds. Cattle were housed at the Mahasarakham University farm, Mahasarakham, Thailand. All cattle were 6 and 8 years old with similar body weight (250–300 kg). Before the study, the cattle were healthy as examined by a veterinarian. They were routinely dewormed and vaccinated. All cattle received concentrate, grazed freely, and had unlimited access to water.

### Experimental design

The design of the experiment consisted of 2 × 4 factorial in a completely randomized design, consisting of eight treatment combinations. The first factor was two breeds: Brahman cattle (n = 3) and Brahman × Thai native crossbreed cattle (n = 3). The second factor was the level of LPS (0, 0.1, 1, and 10 μg/mL). Therefore, the treatment combination used in this experiment was as follows:

Treatment combination; (1) PBMC of Brahman × Thai native crossbreed cattle that received medium control (without LPS stimulation). (2) PBMC of Brahman × Thai native crossbreed cattle that received LPS treatment at 0.1 μg/mL. (3) PBMC of Brahman × Thai native crossbred cattle that received LPS treatment at 1 μg/mL. (4) PBMC of Brahman × Thai native crossbred cattle that received LPS treatment at 10 μg/mL. (5) PBMC of Brahman cattle that received medium control (without LPS stimulation). (6) PBMC of Brahman cattle that received LPS treatment at 0.1 μg/mL. (7) PBMC of Brahman cattle that received LPS treatment at 1 μg/mL. (8) PBMC of Brahman cattle that received LPS treatment at 10 μg/mL.

### Blood collection

A total of 20 mL of blood was aseptically collected from each cow from the jugular vein, transferred into sterile ethylenediaminetetraacetic acid vacutainers and transported to the laboratory within 2 h for isolation of PBMCs.

### Isolation and culture of PBMCs

PBMCs were isolated from whole blood samples using the density gradient centrifugation assay described by Rattanasrisomporn *et al*. [18]. The PBMCs in this investigation were isolated directly from cattle and were comparable to the primary cell lines used to study the different responses to LPS [19, 20]. A 1:2 volume ratio of phosphate-buffered saline (PBS) was used to dilute the blood sample. Subsequently, diluted blood was gently layered on Ficoll-Paque™ Plus (GE Healthcare, Diegem, Belgium) and centrifuged at 800× *g* for 30 min. The intermediate layer containing the PBMCs was carefully collected and washed twice with PBS. Red blood cell lysis buffer (Sigma-Aldrich, St. Louis, MO, USA) was used to lyse the red blood cells. The cells were then resuspended in Roswell Park Memorial Institute (RPMI) 1640 medium containing 10% fetal bovine serum (Sigma-Aldrich) and 1% penicillin-streptomycin (Gibco, USA). After 24 and 48 h of incubation, the morphology of PBMCs was examined using an inverted microscope.

### Cell viability assay

The cell viability assay was performed using an MTT (3-[4,5-dimethylthiazol-2-yl]-2,5 diphenyl tetrazolium bromide) assay described by Rattanasrisomporn *et al*. [18]. Each well of a 96-well plate was seeded with 2.5 × 10^5^ PBMC cells from Brahman and Brahman × Thai native crossbreed cattle and incubated with different doses of LPS (0, 0.1, 1, and 10 μg/mL) at 37°C and 5% CO_2_ for 48 h. After incubation, the supernatant was removed and MTT (5 mg/mL in PBS) (20 μL) was added to each well. The crystalline formazan product was dissolved with 150 μL of dimethyl sulfoxide after 4 h of incubation. The optical density was measured at 570 nm using a microplate reader (Tecan, Switzerland).

### Determination of MDA, NO, and TAC

PBMCs were plated into 12-well plastic tissue culture plates in RPMI 1640 media at a density of 1.5 × 10^6^ cells/mL, resulting in a final volume of 3 mL per well. Subsequently, the PBMCs of Brahman cattle and Brahman × Thai native crossbreed cattle were exposed to different conditions. The control group was not treated with LPS, whereas the other groups were administered increasing doses of LPS (0.1, 1, and 10 μg/mL). Morphological alterations were observed at 2 time points (24 and 48 h) using an inverted microscope (Nikon Corporation, Tokyo, Japan). The supernatant sample at 48 h was kept at −20°C for MDA, NO, and TAC analysis.

MDA levels in the cell culture supernatants were determined after 48 h of LPS exposure using a thiobarbituric acid reactive substance assay described by Srinontong *et al*. [21]. Briefly, aliquots of the reaction mixture containing cell supernatant, 0.12 M thiobarbituric acid, 0.9% NaCl, and 10% trichloroacetic acid were heated at 100°C for 30 min. After cooling, the mixtures were centrifuged at 1100*× g* for 10 min using a Hettich Rotina 380R centrifuge (Germany). A Tecan Infinite® 200 Microplate Reader (Tecan Trading AG, Männedorf, Switzerland) was used to analyze the reaction mixture at 532 nm and compared with the MDA standard (1,1,3,3-tetraethoxypropane).

NO concentration was determined by measuring its end-products, nitrate, and nitrite, using the Griess method described by Csonka *et al*. [22]. A Griess solution containing 1% sulfanilamide and 0.1% (w/v) N-(1-naphthyl) ethylenediamine dihydrochloride in 2.5% (v/v) phosphoric acid was prepared. Next, an equal volume of the Griess reagent and the supernatant were mixed. Following a 15-min incubation at room temperature (25°C), a standard curve was read at 540 nm using a microplate reader (Tecan Trading AG).

TAC of the supernatant was performed using a ferric-reducing antioxidant power (FRAP) assay, as described by Benzie and Strain [23]. The fresh FRAP working solution was prepared by combining FeCl_3 (_20 mM), TPTZ reagent (10 mM 2,4,6-tri-pyridyl-s-triazine solution in 40 mM HCl), and acetate buffer (0.3 M, pH 3.6) in a 1:1:10 ratio. Next, 500 μL of the supernatant was mixed with 7.5 mL of the FRAP working solution. The absorbance at 593 nm was measured using a Tecan Infinite® 200 Microplate Reader after standing for 6 min at 25°C, and a ferric chloride standard curve was used for the calibration. The FRAP value was expressed as mM Fe^2+^ per gram of the sample.

### Statistical analysis

The normal distribution of data was tested. A two-way analysis of variance (Proc Analysis of Variance) was used to analyze the data. Mean values were separated using Duncan’s multiple range tests (SAS® studio, San Diego, CA, USA). The level of significance was set at p < 0.05.

## Results

### Impact of LPS concentration on PBMC cell morphology in Brahman and Brahman × Thai native crossbreed cattle

**Figure-1:**
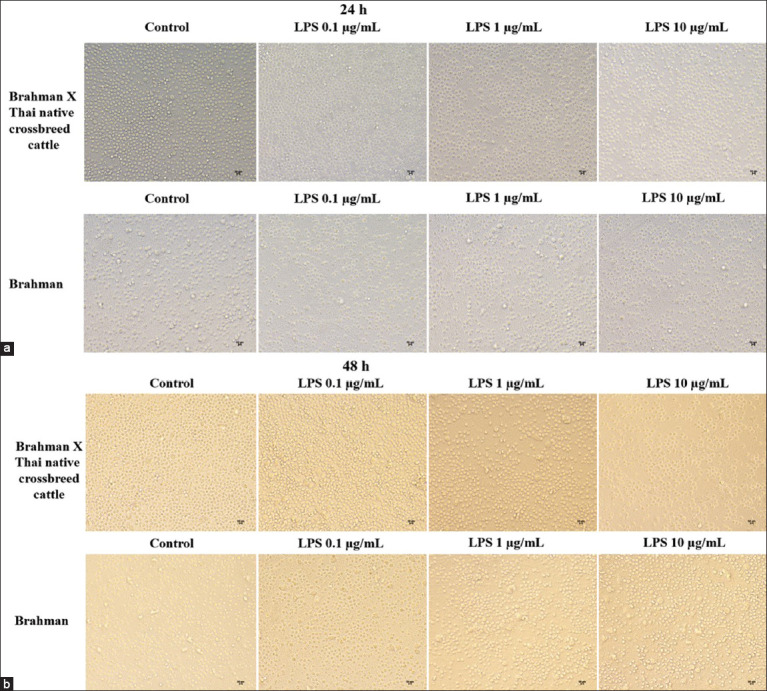
The morphology of PBMC from Brahman and Brahman × Thai native crossbreed cattle after LPS treatment (0, 0.1, 1.0, and 10 μg/mL) for (a) 24 h and (b) 48 h. LPS=Lipopolysaccharide, PBMC=Peripheral blood mononuclear cells.

Compared with the control group, stimulation of PBMCs with varying concentrations of LPS for 24 and 48 h did not result in morphological changes. All groups exhibited round cell morphology after 24 h, and some PBMCs displayed adherent characteristics and enlarged size after 48 h (Figure-1).

### Effect of LPS concentration on PBMC viability, MDA, NO, and TAC of Brahman and Brahman × Thai native crossbreed cattle

Brahman and Brahman × Thai native crossbreed cattle PBMC viability was not significantly different (p > 0.05). PBMC viability after treatment with LPS at 0, 0.1, 1.0, and 10 μg/mL was not significantly different (p > 0.05). NO of PBMC from Brahman cattle was significantly higher than Brahman × Thai native crossbreed cattle (p < 0.05). NO of PBMC treated with LPS at 0, 0.1, 1.0, and 10 μg/mL was not significantly different (p > 0.05). MDA of PBMC from Brahman cattle was significantly higher than Brahman × Thai native crossbreed cattle (p < 0.05). MDA of PBMC that were treated with LPS at 0, 0.1, 1.0, and 10 μg/mL was not significantly different (p > 0.05). The TAC of PBMC from Brahman cattle was significantly lower than Brahman × Thai native crossbreed cattle (p < 0.05). TAC of PBMC that were treated with LPS at 0, 0.1, 1.0, and 10 μg/mL was not significantly different (p > 0.05) (Table-1).

**Table-1:** Effect of LPS on PBMC viability, MDA, NO, and TAC of untreated and LPS-treated bovine PBMCs derived from Brahman and Brahman×Thai native crossbreed cattle.

Parameters	Breed (B)		Concentration of LPS (mg/mL) (C)				p-value		
		
	Brahman× Thai native crossbreed	Brahman	0	0.1	1	10	B	C	B*C
Cell viability (%)	93.62 ± 6.31	82.03 ± 5.58	100 ± 11.36	86.49 ± 12.60	81.85 ± 3.66	84.27 ± 3.05	0.193	0.4194	0.754
NO (μM)	3.11^b^ ± 0.02	3.17^a^ ± 0.01	3.12 ± 0.03	3.15 ± 0.02	3.15 ± 0.02	3.15 ± 0.01	0.0096	0.6283	0.9537
MDA (μM)	21.02^b^ ± 2.16	35.18^a^ ± 3.25	32.87 ± 7.66	30.83 ± 4.01	24.89 ± 4.53	23.43 ± 3.21	0.0012	0.163	0.3628
TAC (mM Fe^2+^/g)	70.29*^a^* ± 2.06	49.49^b^ ± 4.60	56.73 ± 10.19	65.9 ± 4.23	55.26 ± 5.6	59.17 ± 6.01	0.0011	0.581	0.2889

Mean ± SE with different letters within a row, significantly different at p *<* 0.05. B=Breed; C=Concentration of LPS (mg/mL), (B*C)=Interaction effects between breed and concentration of LPS. LPS=Lipopolysaccharide, PBMC=Peripheral blood mononuclear cells, TAC=Total antioxidant capacity, NO=Nitric oxide, MDA=Malondialdehyde, SE=Standard Error

## Discussion

LPS is widely used to induce acute stress in animals. However, different types of animals and species may respond differently to LPS stimulation [24]. Although LPS is widely used, the specific effects of LPS on beef cattle (especially for different breeds and levels of LPS) remain unknown. However, there is still limited and unclear information. Therefore, this study was designed to study how PBMCs from different breeds of cattle respond to LPS using LPS to stimulate PBMCs, which are cells in the immune system. In addition, the influence of LPS at different levels on PBMC morphology, cell viability, MDA and NO production, and TAC levels was also studied.

In general, LPS can cause cell damage because it induces oxidative stress and causes cell death [25]. When stimulated with LPS for 48 h, the size of some PBMC cells was increased. This may be due to the behavior and abilities of cells that can change according to the environment [26]. LPS is a factor that can stimulate an innate immune response [27]. In this study, we used an *in vitro* model to determine whether LPS can change PBMC morphology after stimulation with LPS in beef cattle. We found that some PBMC cells of both breeds of cattle changed their shape after treatment with these substances. This finding is consistent with a study by Haugland *et al*. [28], who reported the effects of various mitogens, including LPS, on the mononuclear blood cells of Atlantic salmon (*Salmo salar* L.) and found that LPS caused their mononuclear blood cells to undergo morphological changes. The cell viability of PBMCs in both cattle breeds was not different. The results of this study are consistent with those reported by Amadori *et al*. [29], who found no change in the viability of PBMCs of cattle exposed to different levels of LPS. The results of the present study were similar to those of studies on chicken PBMCs [18] and human PBMCs [30], which found that LPS did not affect the viability of these cells.

However, it was found that the breed of beef cattle affects the level of oxidative stress. The MDA of PBMCs in Brahman cattle was higher than that in Brahman × Thai native crossbreed cattle. These results suggest that after stimulation with LPS, Brahman cattle had higher levels of oxidative stress than Brahman × Thai native crossbreed cattle. Our findings are consistent with those of De Matteis *et al*. [31], who reported that oxidative stress levels were lower in Simmental × Holstein crossbreed cattle than in pure breed parents. Gonzalez-Añover *et al*. [32] found that plasma H_2_O_2_ concentrations were lower in lean hybrid pigs than in purebred Iberian pigs. However, Limousin cattle (imported breed) have lower levels of oxidative stress than Qinchuan cattle (local breed) in China [33]. Therefore, this study demonstrates that cattle breed influences oxidative stress.

Moreover, the NO level of PBMCs in Brahman cattle was found to be higher than that in Brahman × Thai native crossbreed cattle after incubation with LPS. The results of the present study are consistent with those of Silanikove *et al*. [34], who used bovine epithelial cells.

When MDA and NO levels were measured, MDA and NO levels of PBMCs from Brahman cattle were higher than those from Brahman × Thai native crossbreed cattle. In addition, increases in MDA and NO levels indicated an imbalance between free radicals and antioxidants. Therefore, when measuring the level of TAC in the supernatant, it was found that the TAC of Brahman × Thai native crossbreed cattle was higher than that of Brahman cattle. The higher TAC levels in Brahman × Thai native crossbred cattle than in Brahman cattle were in line with the increase in MDA and NO levels in Brahman cattle, which were higher than those in Brahman × Thai native crossbred cattle. The results of the present study are consistent with those of studies on other animals, such as the fact that TAC levels in Italian saddle horses are higher than those in northern Italian thoroughbred horses. This suggests that genetic variation affects TAC levels [35]. In contrast, Mann *et al*. [36] found that during the early and peak stages of lactation, TAC levels were lower in milk from Sahiwal cows (indigenous Indian cattle) than in milk from Karan Fries cows (hybrids). Our results suggest that the immune cells of Brahman × Thai native crossbreed cattle have better antioxidant capacity than those of Brahman cattle, indicating that crossbreed cattle are more resistant to stress responses and oxidative stress than purebred breeds.

## Conclusion

Morphological changes in cell viability, MDA, NO, and TAC of PBMCs isolated from Brahman and Brahman × Thai native crossbreed cattle after stimulation with different levels of LPS were studied. LPS affected the morphology of PBMCs but did not affect the viability of PBMCs. MDA and NO levels in PBMCs isolated from Brahman cattle were higher than those isolated from Brahman × Thai native crossbreed cattle. On the other hand, the TAC of Brahman × Thai native crossbreed cattle was higher than that of Brahman cattle. This study shows that native crossbreed beef cattle are more resistant to oxidative and nitrosative stress than purebred beef cattle. However, *in vitro* studies of LPS-mediated activation of PBMCs do not fully indicate the *in vivo* effects of LPS. Therefore, further studies using animal models are required for further investigation.

## Authors’ Contributions

PS and WA: Designed and performed the experiment and drafted and revised the manuscript. SS, SK, TN, and BS: Data analysis and the fieldwork. TC and WS: Reviewed the manuscript and data analysis. ZW: Data interpretation, edited the manuscript and performed final manuscript revision. All authors have read, reviewed, and approved the final manuscript.
